# Thyroglossal duct cyst carcinomas — a retrospective study and systematic review of the literature

**DOI:** 10.1007/s00428-025-04125-2

**Published:** 2025-05-10

**Authors:** Vivian Thimsen, Sarina Katrin Müller, Abbas Agaimy, Konstantinos Mantsopoulos, Michael Beck, Michael Koch, Heinrich Iro, Matti Sievert

**Affiliations:** 1 Department of Otorhinolaryngology, Head and Neck Surgery, Friedrich Alexander University of Erlangen–Nuremberg (FAU), Waldstrasse 1, 91054 Erlangen, Germany; 2 Department of Pathology, Erlangen University Hospital, Friedrich Alexander University of Erlangen– Nuremberg, Erlangen, Germany; 3 Department of Nuclear Medicine, Erlangen University Hospital, Friedrich Alexander University of Erlangen–Nuremberg, Erlangen, Germany

**Keywords:** Thyroglossal duct cyst, Carcinoma, Papillary, Treatment algorithm

## Abstract

The aim of this study was to analyze thyroglossal duct cyst carcinoma (TDCC) in a single-center retrospective analysis, supplemented by a systematic literature review to inform treatment approaches. Patient records from a tertiary referral center were analyzed for individuals diagnosed with TDCC between 2002 and 2023. A systematic review followed the PRISMA guidelines, encompassing studies from Medline and PubMed. Patient data, including demographics, imaging results, and histological findings were extracted. The primary outcome assessed was tumor-free and recurrence-free survival, while additional variables included treatment regimens and follow-up data. The analysis identified a total of 484 TDCC cases, confirming papillary thyroid carcinoma (PTC) as the predominant type (94.2%), with synchronous thyroid gland carcinomas observed in 34.6%. The age range was 8 to 76 with median age of 40 years. Women were affected in 62%, men in 36%. The recurrence rate was 7.4%, with distant metastases observed in 1% of cases. The overall survival rate was 99.1%, regardless of the treatment regimen. Although rare, TDCC predominantly presents as PTC, with a favorable prognosis. Sistrunk’s procedure remains the primary surgical approach, but optimal management regarding therapies such as total thyroidectomy, neck dissection, or radioiodine ablation requires careful evaluation of risk factors like rare tumor types, suspicious lymph nodes, thyroid nodules, age, radiation exposure, and molecular patterns. Emphasizing more individualized treatment strategies, we propose an algorithm that can help to reduce invasiveness and overtreatment risks in selected cases.

## Introduction

Thyroglossal duct cysts (TDCs) are embryonic remnants formed during thyroid development, as the thyroid gland descends from the foramen cecum to its final position. This congenital anomaly, which persists in about 7% of adults, is slightly more common in females [1–4]. Normally, the thyroglossal duct obliterates by the tenth week of gestation [1]. TDCs are usually benign, although about 1% may show malignant transformation of one of its epithelial derivatives. Malignancy originating within TDCs was first described by Brentano in 1911 [5, 6].

Thyroglossal duct cyst carcinoma (TDCC) often resembles benign TDC clinically, with 95.1% being asymptomatic; only a small fraction of patients experiences pain (1.8%) or dysphagia (3.1%) [7]. Diagnostic differentiation using ultrasound (US) is difficult, as carcinomas may sometimes appear as distinct lesions within the cyst [8]. Malignancy indicators — such as irregularity, firmness, invasive behavior, or lymphadenopathy — typically emerge only in advanced cases. Regional lymph-node metastasis is rare, occurring in 2.0–7.7% of cases [9, 10]. Fine-needle aspiration (FNA) is unreliable for diagnosis, as cysts are often hypocellular due to their high fluid content, resulting in a low sensitivity rate of 50–60% [11].

Histologically, approximately 80% of TDCC cases are papillary thyroid carcinomas (PTCs), with rarer types including follicular (FTC), and poorly differentiated (PDTC) thyroid carcinoma, and squamous cell carcinoma (SCC) [12–14]. Medullary thyroid carcinoma (MTC) is unlikely in TDCs, as the cyst epithelium represents almost exclusively follicular cells [1, 15, 16]. Only one study has documented neuroendocrine cells in TDCs [17]. Thyroid tissue is present in the cyst walls in two-thirds of cases, supporting the theory that TDCC may develop as a primary neoplasm, although metastasis from an occult thyroid carcinoma remains a possibility [9, 15, 18]. Widström et al. outlined four criteria for primary TDCC: no malignancy in the thyroid, carcinoma location in the cyst wall or within cyst lumen, squamous or cylindrical epithelium in the cyst wall, and normal thyroid follicles in the cyst [18]. A study by Bakkar et al. found a 43% risk of synchronous thyroid cancer in patients with normal thyroid US findings, although the clinical relevance of this is uncertain [19].

The rarity of TDCC and the lack of randomized studies have hindered the development of standardized treatment protocols. This challenge underscores the difficulty of balancing effective disease control with the risks of overtreatment, such as total thyroidectomy (TT), neck dissection (ND), and adjuvant radioactive iodine (RAI) ablation. The aim of the present study was therefore to review TDCC cases in a single-center retrospective analysis alongside a systematic literature review, in order to establish a reliable treatment approach.

## Materials and methods

This study was conducted at an academic tertiary referral center specializing in head and neck surgery. Informed consent was obtained from each patient for diagnostic, therapeutic, and data processing procedures, all of which were approved by the university’s ethical review board. The study adhered to the university’s general contract conditions and the World Medical Association Declaration of Helsinki.

The records for all patients treated for suspected thyroglossal duct cysts (TDCs) between January 2002 and October 2023 were analyzed retrospectively. The primary aim was to determine the proportion of primary malignancies within these cysts and evaluate the treatment regimens employed. Surgical reports were thoroughly reviewed, and all histological findings were reevaluated by a specialized head and neck pathologist. Patient-related data collected included age, gender, chronic diseases, prior treatments (such as radiotherapy), preoperative imaging modalities (ultrasound, magnetic resonance imaging, computed tomography) and preoperative histology (fine-needle aspiration and core needle biopsy). For patients with confirmed thyroglossal duct carcinoma (TDCC), tumor staging, the presence of simultaneous thyroid cancer, adjuvant therapy, and follow-up results were assessed.

**Fig. 1:**
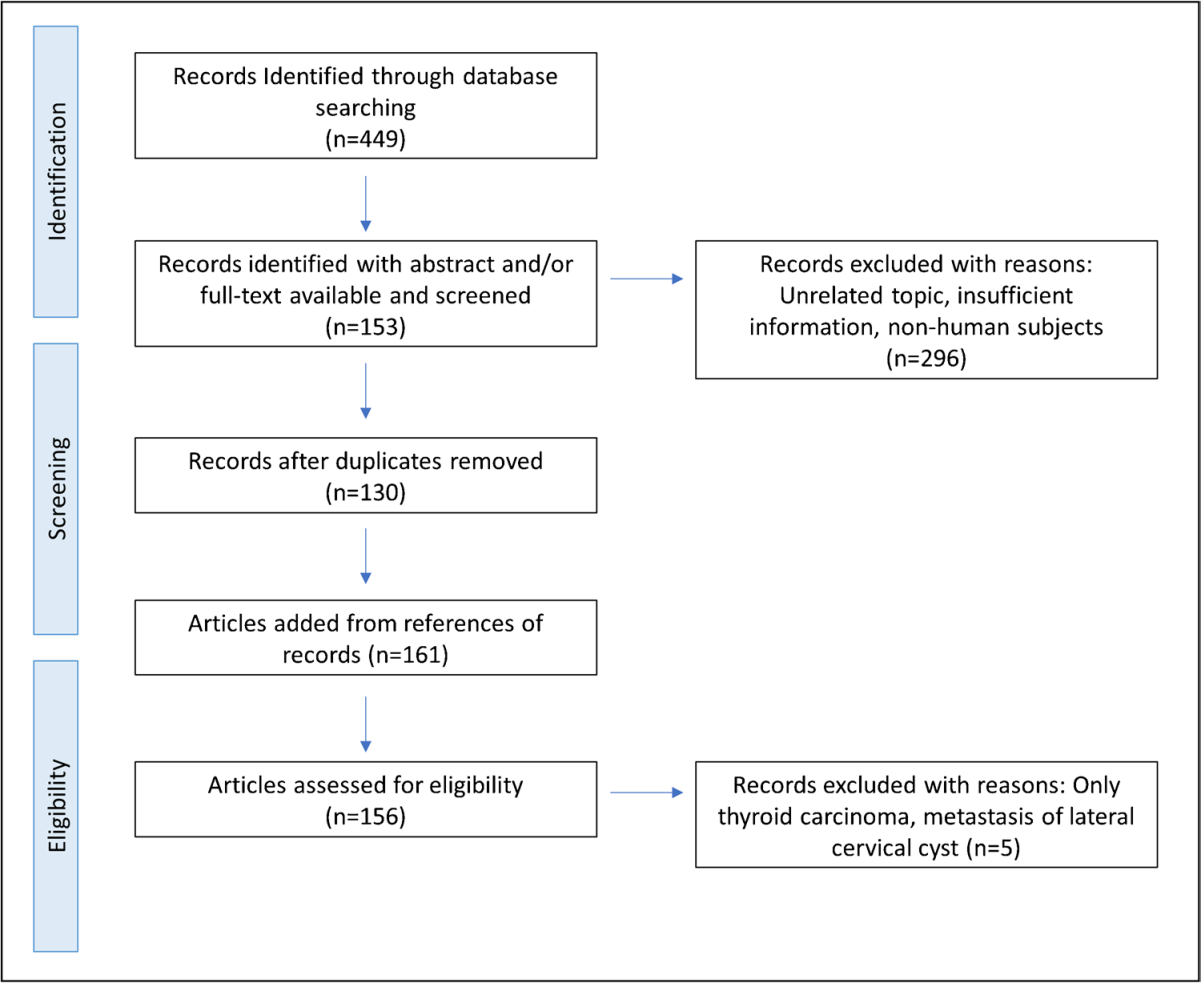
Article selection process, based on the Preferred Reporting Items for Systematic reviews and Meta-Analyses (PRISMA)

Additionally, a systematic literature review was conducted following the PRISMA guidelines (Fig. 1) [20]. A comprehensive search of the Medline and PubMed databases was performed to identify relevant studies, using the search terms “thyroglossal duct cyst” AND “carcinoma” OR “malignant” OR “cancer” OR “papillary carcinoma.” The titles and abstracts of the studies identified were screened for eligibility, with the inclusion criterion being histological confirmation of malignancy in thyroglossal duct cysts. Full-text articles of eligible studies were retrieved and reviewed in detail. This included case reports, case series, retrospective studies, and reviews focusing on malignant tumors in thyroglossal duct cysts. The primary outcomes assessed were tumor-free survival and recurrence-free survival. Additionally, the reference lists of the studies included were manually searched to identify further relevant studies.

Data extraction was performed independently by two reviewers (VT and MS) using a standardized form. Extracted data included author(s), year of publication, number of patients with TDCCs, gender, age, preoperative imaging modalities, histological evaluation, tumor type, surgical approach, adjuvant therapy, follow-up duration, and recurrence rates. Discrepancies between the reviewers were resolved through discussion. To avoid selection bias, no study was excluded due to missing data; only patients with available data for each parameter were included, with the total patient count being adjusted to mitigate distortion and attrition bias.

The primary source of bias in this review stems from the inclusion of case reports and series, which lack randomization and control. However, this limitation is unavoidable due to the rarity of TDCCs, as retrospective analyses of previously published data cannot fully assess performance and reporting bias.

## Results

### Data analysis

Between January 2002 and October 2023, 414 patients with the ICD-10 diagnosis code Q18.8 were evaluated. Following a review of surgical and histological records, 62 patients were excluded due to miscoding, leaving 352 patients (189 female, 163 male, average age 37 years [range: 1–82 years]) for analysis. All of the patients underwent preoperative US, with magnetic resonance imaging (MRI; *n* = 60) or computed tomography (CT; *n* = 23) conducted in selected cases. Four patients had prior head and neck radiation exposure. The Sistrunk procedure, involving resection of the cervical mass and the hyoid bone, was performed on all patients. Among these, 327 were primary operations, while 14 were revision operations in patients referred following previous procedures that had not involved hyoid bone resection.

**Fig. 2:**
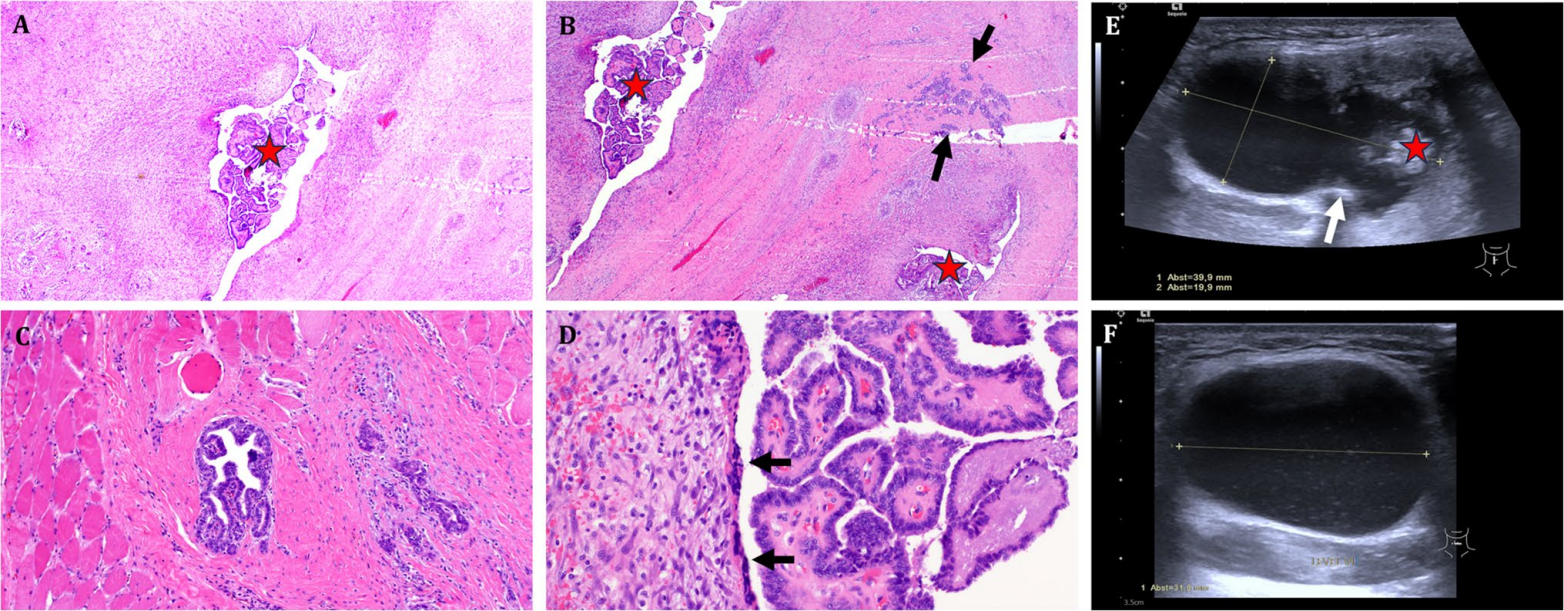
Representative example of imaging and histopathology of TDCC.** A**: Overview of a TDC showing cyst lumen lined by attenuated epithelium and filled by exophytic papillary tumor growth (PTC; red star). **B**: another area showing the intraluminal PTC (red stars) in addition to locules of atrophic thyroid tissue/follicles (black arrows). **C**: infiltrating intramural foci of PTC surrounded by normal skeletal muscle tissue indicating separation of the tumor from the thyroid gland. **D**: higher magnification of the PTC, note attenuated epithelium lining the TDC wall on left (arrows). **E**: Ultrasound image in sagittal view of one patient with TDCC with typical contact to the hyoid bone (white arrow) and solid parts within the cyst (red star). **F**: Ultrasound image in horizontal view of the same patient with TDCC

Histopathological examination identified five cases of TDCC, representing 1.4% of the sample (two females, three males, average age 43 years [range: 15–58 years]), all classified as PTC of conventional subtype. In line with tumor board recommendations, three patients underwent TT and central ND, followed by RAI. Preoperative US identified thyroid nodules in two cases; however, histology did not reveal primary carcinoma within the thyroid gland. Another patient, a 58-year-old woman with Hashimoto’s disease, received RAI without undergoing TT. For a 15-year-old boy, a wait-and-scan approach was adopted due to normal US findings in the thyroid and cervical lymph nodes. No complications, such as hypoparathyroidism or vocal cord dysfunction, were observed postoperatively. The median follow-up period was 70 months (range: 11–182 months), with no recurrences reported. Detailed patient data are provided in Table 1. Figure 2 shows the imaging and histologic findings of one typical patient with TDCC.

**Table 1:** Patient data from the retrospective analysis (Erlangen University, 2002–2023)

No	Gender	Age	Entity/Subtype	TNM	SP	TT	CND	RAI	TS	FU (mo)	R
1	F	37	PTC, CV	pT1a pN0 L0 V0 Pn0 cM0	Yes	Yes	Yes	Yes (3930 MBq I^131^)	Yes	170	No
2	F	58	PTC, CV	pT1b cN0 L0 V0 Pn0 cM0	Yes	No	No	Yes (3.7 GBq I^131^)	Yes	146	No
3	M	51	PTC, CV	pT2 pN0 L0 V0 Pn0 cM0	Yes	Yes	Yes	Yes (3.7 GBq I^131^)	Yes	70	No
4	M	53	PTC, CV	pT1 pN1a L0 V0 Pn0 cM0	Yes	Yes	Yes	Yes (3847 MBq I^131^)	Yes	31	No
5	M	15	PTC, CV	pT1b pN0 (0/1) L1 V1 Pn0 cM0	Yes	No	No	No	Yes	5	No

*PTC* papillary thyroid carcinoma, *CV* conventional Subtype, *SP* Sistrunk’s procedure, *TT* total thyroidectomy, *CND* central neck dissection, *RAI* radioactive iodine (therapy); *TS* thyroid hormone substitution; *FU* follow-up (months); R = recurrence

### Systematic literature review

The literature review retrieved 330 case reports, 112 reviews, and seven systematic reviews. After the removal of irrelevant or duplicate articles on the basis of the predefined inclusion criteria, 156 studies from 1974 to 2023 were included for analysis, supplemented by relevant references from selected articles. Among these, 137 were case reports or series (some containing brief literature reviews), eight were retrospective single-center studies (with patient numbers ranging from five to 26), and 11 were pure reviews, one of which was a systematic review. The case reports and single-center studies included are listed in Table 2 [21–23]. Access or language limitations restricted the analysis of four articles to abstracts only. These reports, along with the data from the present study, created a large cohort of 484 TDCC patients (301 females, 172 males, 11 of unknown gender, average age 39.3 years [range: 8–76 y, IQR: 18.8 y]; see Table 3).

**Table 2:** Case reports and single-center studies included

Author	Year	TDCC*	Female	Male	Age
LiVolsi et al	1974	7	3	4	39.3
Page et al	1974	6	4	2	30.5
Joseph et al	1975	2	1	1	46.5
Saharia et al	1975	2	1	1	67.5
Widström et al	1976	1	1	0	18
Trail et al	1977	2	2	0	31
Turner et al	1978	1	1	0	12
Benveniste et al	1980	1	0	1	75
White et al	1982	1	0	1	61
Roses et al	1983	4	2	2	40
Banerjee et al	1986	1	1	0	45
Ronan et al	1986	2	2	0	28
LaRouere et al	1987	2	2	0	24.5
Borger et al	1988	1	1	0	12
McNicoll et al	1988	1	0	1	9
Vincent et al	1989	1	0	1	19
Kashkari et al	1990	1	0	1	65
Pacheco-Ojeda et al	1991	5	5	0	34
Fernandez et al	1991	10	9	1	NA
Weiss et al	1991	1	0	1	41
Adler et al	1991	1	1	0	56
Maziak et al	1992	3	1	2	54
Yanagisawa et al	1992	1	0	1	65
Chen F. et al	1993	3	3	0	38.6
Chen K. et al	1993	2	0	2	43
Kum et al	1993	1	1	0	51
Grabowska et al	1993	1	0	1	33
Van Vuuren et al	1994	3	2	1	33.6
Lieberum et al	1994	3	1	2	38
Mahnke et al	1994	1	0	1	63
Tew et al	1995	4	0	4	53
Bardales et al	1996	3	0	3	54
Hanna et al	1996	1	0	1	50
Ikeda et al	1996	1	0	1	50
Kojima et al	1996	1	1	0	34
Kwan et al	1996	1	0	1	38
Ranieri et al	1996	1	0	1	68
Heshmati et al	1997	12	6	6	40
Asakage et al	1997	1	1	0	47
Walton et al	1997	1	0	1	71
O'Connell et al	1998	8	3	5	44.6
Kennedy et al	1998	4	0	4	39
Yoo et al	1998	1	1	0	11
Kurzen et al	1999	1	0	1	22
Martins et al	1999	1	1	0	21
Datar et al	2000	1	0	1	35
Tradati et al	2000	4	2	2	42.8
Rosoff et al	2000	1	0	1	8
Yang et al	2000	1	0	1	30
Moncet et al	2001	3	1	2	37
Montero Garcia et al	2001	1	1	0	31
Doshi et al	2001	14	8	6	39.3
Falconieri et al	2001	2	1	1	34.5
Samara et al	2001	1	1	0	43
Cignarelli et al	2002	3	0	3	47.3
Patel et al	2002	5	3	2	45
Baysungur et al	2002	1	1	0	40
Chu et al	2002	1	1	0	31
Aluffi et al	2003	2	2	0	48.5
Naghavi et al	2003	1	0	1	28
Öztürk et al	2003	1	0	1	11
Yamada et al	2003	1	1	0	35
El Bakkouri et al	2004	1	1	0	55
Miccoli et al	2004	18	14	4	21–67
Luna-Ortiz et al	2004	5	3	2	49
Peretz et al	2004	1	1	0	15
Fálvo et al	2006	4	3	1	32.8
Plaza et al	2006	5	5	0	28.6
Koybasioglu et al	2006	1	1	0	63
Lehnerdt et al	2006	1	0	1	51
Banerjee et al	2007	2	2	0	14.5
Berni Canani et al	2008	1	1	0	35
Guzman et al	2008	2	2	0	21
Kermani et al	2008	1	1	0	76
Hartl et al	2009	18	13	5	41.5
Aghaghazvini et al	2009	1	1	0	44
Baizri et al	2009	1	0	1	45
Hofmann et al	2009	1	1	0	38
Park et al	2010	1	1	0	46
Mesolella et al	2010	1	0	1	27
Ogawa et al	2010	1	1	0	61
Torcivia et al	2010	2	1	1	53.5
Forest et al	2011	9	NA	NA	44
WangY. et al	2011	3	3	0	40.6
Wang C. et al	2011	1	0	1	51
Albayrak et al	2011	1	0	1	39
Balalaa et al	2011	1	0	1	31
Gomi et al	2011	1	1	0	11
Kinoshita et al	2011	1	1	0	61
Dzodic et al	2012	12	9	3	40.6
Dan et al	2012	1	1	0	18
Schneider et al	2012	1	0	1	47
See et al	2012	1	1	0	39
Yü et al	2012	5	0	5	41
Pellegriti et al	2013	26	20	6	41.23
Choi et al	2013	10	8	2	54
Chrisoulidou et al	2013	6	4	2	39.2
Senthilkumar et al	2013	1	0	1	52
Tharmabala et al	2013	1	1	0	32
Yamada et al	2013	1	1	0	74
Rossi et al	2014	9	5	4	46
Aculate et al	2014	1	1	0	21
Pfeiffer et al	2014	1	1	0	15
Proia et al	2014	1	1	0	20
Vassilatou et al	2014	1	0	1	19
Shah et al	2015	1	0	1	47
Akram et al	2016	6	5	1	41
Chala et al	2016	6	5	1	48.8
Cheon et al	2016	1	1	0	17
Patel N. et al	2016	2	NA	NA	NA
Shemen et al	2016	1	0	1	53
Bakkar et al	2017	19	15	4	58
Thompson et al	2017	22	17	5	40
Naik et al	2017	1	1	0	47
Roehlen et al	2017	1	1	0	21
Sachpekidis et al	2017	1	0	1	69
Alatsakis et al	2018	1	1	0	27
Das et al	2018	1	0	1	36
Wood et al	2018	5	4	1	38
Zhang et al	2018	1	1	0	57
Bakkar et al	2019	15	12	3	55
Liaw et al	2019	1	0	1	15
Korbi et al	2019	1	1	0	14
Moreno et al	2019	1	0	1	63
Van der Heide et al	2020	1	1	0	53
Bahar et al	2020	2	2	0	35.5
Boyanov et al	2020	1	1	0	17
George et al	2020	1	1	0	49
Henry et al	2020	1	1	0	62
Mahmoud et al	2020	1	1	0	31
Puccini et al	2020	1	1	0	45
Ozturk et al	2021	1	1	0	20
Pandey et al	2021	1	1	0	11
Rumman et al	2021	1	0	1	23
Saavedra-Leveau et al	2021	1	0	1	37
De Sousa et al	2022	1	0	1	45
Wong et al	2022	6	4	2	46.8
Rovira et al	2023	12	6	6	46
Bishop et al	2023	8	3	5	40.6
Huang et al	2023	1	0	1	39
Mittal et al	2023	1	0	1	37
Mylopotamitaki et al	2023	1	1	0	16
Solis-Pazmino et al	2023	1	1	0	NA
Suresh et al	2023	30	21	9	33
Zhou et al	2023	1	0	1	24
*This study’s data*	2024	5	2	3	43

^*^TDCC = thyroidal duct cyst carcinoma

**Table 3:** Summary of data for the patients included

Characteristic	Totals	Range
Patients (*n*)	484	
Age at diagnosis (y)	39.27	8–76
Sex, male/female (%)	62/36*	
Histopathological entity		
PTC	456	
FTC	5	
Oncocytic FTC	4	
SCC	15	
ASC	1	
PDTC	1	
Recurrences (%)	7.4	
Locoregional	5.9	
Distant	1.4	
Deaths (%)	0.95	
Mean follow-up (mo)	40.62	0–4

^*^ Gender data were missing for 2% of the patients. PTC = papillary thyroid carcinoma; FTC = follicular thyroid carcinoma; SCC = squamous cell carcinoma; ASC = adenosquamous carcinoma; PDTC = poorly differentiated thyroid carcinoma

PTC was the most prevalent type of TDCC, present in 94.2% (456 cases), with other tumor types comprising SCC (*n* = 15), FTC (*n* = 5), oncocytic (former “Hürthle cell”) FTC (*n* = 4), adenosquamous carcinoma (ASC, *n* = 1), and PDTC (*n* = 1). Two patients had concurrent PTC and SCC within the thyroglossal duct cyst [13, 24]. Due to the systematic review, we were dependent on the histopathological description of the primary source, which explains some of the old nomenclature used in the manuscript. Table 4 outlines tumor size, lymph node involvement, and metastasis status. Given the lack of specific staging criteria for TDCCs, the tumors were classified retrospectively in accordance with the International Union Against Cancer (UICC) thyroid carcinoma guidelines (eighth edition), depending on the data provided. Preoperative diagnostic data are detailed in Table 5.

**Table 4:** Distribution of TNM classifications among the patients included

Tumor size		Lymph nodes		Distant metastases (initial)	
pT1	119 (24.6%)	N0	313 (64.7%)	cM0	286 (59.0%)
pT1a	72 (14.9%)	cN0	288 (59.5%)		
pT1b	31 (6.4%)	pN0	25 (5.2%)		
NA	16 (3.3%)				
pT2	18 (3.7%)	N +	82 (16.9%)	cM1°	3 (0.6%)
		DTC pN1a	22 (4.5%)		
		DTC pN1b	57 (11.8%)		
		Other pN + ^n^	3 (0.6%)		
pT1 + pT2*	178 (36.8%)	Nx	89 (18.4%)	cMx	195 (40.3%)
pT3	57 (11.8%)				
pT4	5 (1.0%)				
pTx	244 (50.4%)				

*DTC* differentiated thyroid carcinoma

^*^ Differentiation between pT1b and pT2 was not possible due to missing information in 41 patients; these patients were therefore added to the pT1 + pT2 section in addition to the patients with these tumor size categories listed above

° Two oft three patients with distant metastasis had SCC, one of whom had simultaneous laryngeal carcinoma

^n^ Two patients with squamous cell carcinoma within the thyroglossal duct cyst were classified according to the SCC in the head and neck classification as pN1 and pN2b

**Table 5:** Preoperative diagnostic data for the patients included

Diagnostic method*	Data available	Examinations performed
US	443/484	370 (83.5%)
CT	361/484	112 (31.0%)
MRI	361/484	26 (7.2%)
FNA	420/484	203 (48.3%)
True-positive		122 (60.1%)
False-negative		79 (38.2%)
Sensitivity		60%

^*^US ultrasound; *CT* computed tomography; *MRI* magnetic resonance imaging; *FNA* fine-needle aspiration

### Surgical treatment

Surgical intervention was performed for diagnostic confirmation in all cases. The Sistrunk procedure with partial hyoid bone resection was used in 95.6% of cases (457/478), while TT was conducted in 66.4% following a TDCC diagnosis (318/479). Thyroid abnormalities were identified preoperatively in only 25.6% of cases (68/266). In 0.8% (4/479), limited thyroid surgery (e.g., lobectomy) was performed to exclude malignancy in patients with suspicious thyroid nodules. Simultaneous thyroid carcinoma was observed in 34.6% of patients with TT (110/318), primarily PTC (95.5%). Two cases involved mixed PTC/FTC (1.8%), two were FTC (1.8%), and one revealed concomitant medullary carcinoma (MTC, 0.9%) [19, 25–29]. Notably, 6.4% (7/110) of these cases presented differing tumor types in the TDC and thyroid gland. Among the 105 cases of PTC, 40.9% were smaller than 1 cm (T1a, ≤ 1 cm), with only two showing extra-thyroid extension (T3, 1.9%). Other cases were staged as T1b or T2, with no T4 tumors detected. Additionally, 34.5% (38/110) showed multilocular carcinoma within the thyroid. Notably, 39.6% (19/48) of simultaneous thyroid carcinomas were found in patients with preoperatively regular US scans; 47.4% (9/19) of the lesions were smaller than 1 cm.

### Lymph-node involvement

Central ND was performed in 23.2% (96/414) and lateral ND in 15.7% (65/415) of the patients. During follow-up, two patients (0.5%) required lateral ND for suspicious lymph nodes. Lymph-node metastases were reported in 20.9% (82/393) of cases, with 26.8% (22/82) found in the central compartment (pN1a, all differentiated thyroid carcinoma) and 73.2% (60/82) in the lateral compartment. Most lateral metastases were differentiated thyroid carcinoma (57 cases, pN1b), with three involving SCC (other pN +). In patients with central lymph-node metastases, 50% (11/22) had concurrent thyroid carcinoma, in comparison with 36.8% (21/57) among those with lateral metastases. Tumor size correlated with local metastasis, with larger tumors (pT3 or greater) more frequently presenting with lymph-node involvement: 43.8% (7/16) of patients with pN1a and 36.4% (12/33) with pN1b had tumors classified as pT3 or more. In contrast, only 9.42% (11/119) of patients with pT1 tumors and 10.53% (2/19) with pT2 tumors had locoregional metastases (pN1a/b).

### Distant metastasis

Distant metastases (M1) were rare, found in only 1.0% (3/289) of cases. Two patients with SCC had distant metastases, one of whom also had a synchronous laryngeal carcinoma. Only one PTC patient presented with distant metastasis in the lung at diagnosis [30].

### Adjuvant treatments and follow-up

RAI was administered in 48.7% of cases (220/452); 96.8% (213/220) of these patients received RAI after TT. Only three patients received RAI without prior TT. Dosage information was available for 41 patients, with a median total dose of 3.7 GBq (range: 1.0–9.0 GBq). External beam radiation therapy (EBRT) was used in 3.6% (15/411) of cases, primarily in patients with SCC (73.3%, 11/15) in the TDC or with concurrent laryngeal carcinoma (6.1%, 1/15). In some patients with PTC within the TDC, RAI was also used (20%, 3/15). The median EBRT dose was 60 Gy (range: 50–66 Gy).

The mean follow-up period was 41.53 months (range: 2–224 months, IQR = 46.1), with data available for 385 patients. In 7.4% (31/421) of the patients, recurrences were observed as locoregional lymph-node metastases (25/31). Twenty-three of the patients had PTC (92.0%); of these, eight had concurrent papillary thyroid carcinoma, and two suffered from SCC (8.0%). Six patients experienced distant metastases (five PTC, one SCC), primarily in the lung. The mortality rate attributed to TDCCs was 0.95% (4/422; one SCC, three PTCs), with three deaths associated with distant metastases (one SCC, two PTCs). None of the patients with distant metastases or death by the disease had simultaneous orthotopic thyroid carcinoma.

## Discussion

TDCs make up around 7% of adult malformations that are clinically relevant, especially in individuals under 30 years of age. Malignancies in these cysts are rare (approximately 1%), in patients with a median age of 40 years, affecting females almost twice as frequently [3, 4, 6, 7]. This pattern aligns with primary papillary thyroid carcinomas (PTCs), which are the dominant histotype of TDCC, at 96%.

### Diagnostic considerations

Ultrasonography is the preferred initial diagnostic tool for assessing neck masses, valued for its high degree of accuracy and accessibility. It can reveal malignancy indicators such as solid components or invasive growth [8, 31]. FNA is useful for lesions with solid elements, especially in the presence of thyroid nodules, though the sensitivity of FNA for TDCC is approximately 60% [11, 32, 33]. Some authors recommend at least three punctures to improve accuracy [11, 34]. Tumor markers specific to TDCC have not been established, although elevated thyroglobulin levels in blood or cysts may indicate malignancy [35]. However, these findings should be interpreted cautiously, as elevated thyroglobulin levels can also occur in inflammatory conditions such as Hashimoto’s thyroiditis, and their diagnostic value becomes relevant only after total thyroidectomy [1]. There is also a lack of comparative data from benign TDC aspirates (with or without inflammation).

Cross-sectional imaging is advised for patients with positive nodal status (pN +) or suspected invasive growth, to evaluate areas such as the larynx or trachea.

### Histopathology of TDCC

PTC accounts for nearly 95% of TDCC cases and therefore leads the recommended treatment protocols and defines outcomes. Rarer entities such as SCC, FTC, HCC, ASC, and PDTC require tailored approaches. A key topic of debate in the literature concerns the origin of PTC in TDCC, with two main theories: de novo tumor formation versus metastasis from a primary thyroid carcinoma [9, 15, 36].

The data in the present study show a 34.6% concurrence of orthotopic thyroid carcinoma with TDCC in patients who underwent additional TT, implying that solitary TDCC is present in 76.0% of all patients and 65.4% of patients with TT. 90.0% of the patients with simultaneous thyroid gland carcinoma revealed PTC both in TDC and in the proper gland. Therefore, 54.4% of the thyroid gland carcinomas were < 10 mm and 41.4% occurred multifocal within the gland. 11 cases (10.0%) revealed different histopathological entities in TDC and thyroid gland. In six cases a classic variant of PTC was found within the TDC whereas the foci within the thyroid gland showed a follicular variant of PTC. Two cases revealed PTC within the TDC and FTC within the thyroid gland and again two cases showed FTC within the TDC and PTC within the thyroid gland. In one patient, however, a simultaneous MTC was found within the thyroid gland, while TDC showed a PTC. Evidence against the metastasis hypothesis includes the presence of thyroid parenchyma in two-thirds of the cysts, and the absence of medullary thyroid carcinoma in TDCC [9, 15]. In addition, patients with genotypically distinct tumor entities in the thyroid and TDCC with synchronous carcinomas further support the hypothesis of de novo tumor formation [26, 28]. This suggests that tumor formation may occur in thyroid cells present in the cyst wall. The occurrence of SCC in the thyroid is considered a pattern of anaplastic thyroid carcinoma (ATC) and this histological type may occur combined with PTC, other ATC patterns or as pure SCC. In TDCs, SCC however can conceivably originate from the lining squamous epithelial elements of the cyst, in the sense of genuine primary SCC, but limited data does allow for any conclusive statement on pathogenesis and inherent prognosis of pure SCC originating within TDC.

### Therapeutic decision-making

**Fig. 3:**
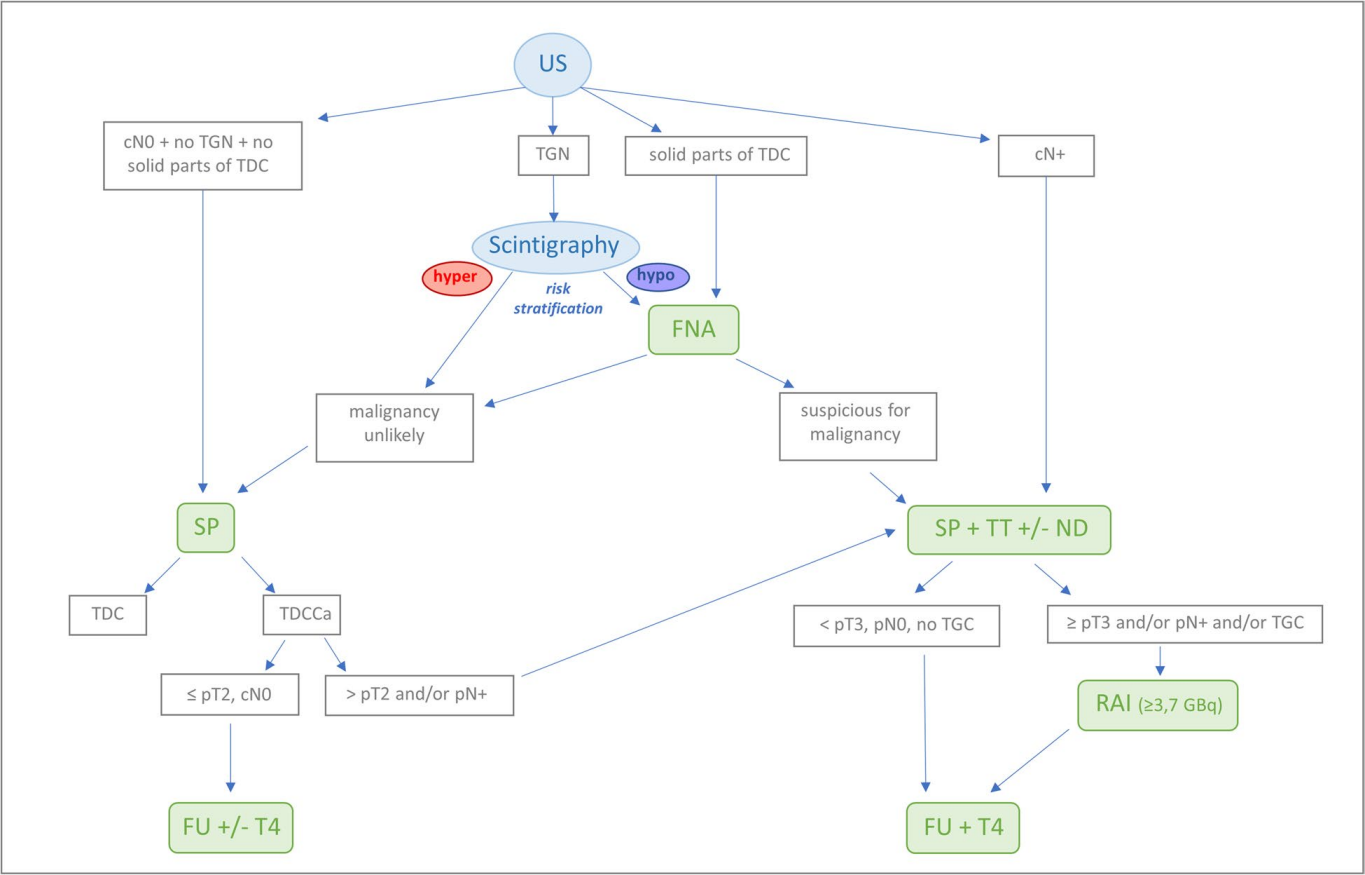
Algorithm for the treatment of differentiated thyroid gland carcinoma within a thyroglossal duct cyst. cN +, clinical positive nodal status; FNA, fine-needle aspiration; FU, follow-up; ND, (bilateral) neck dissection; pN +, histopathologic positive nodal status.; RAI, radioiodine therapy; SP, Sistrunk procedure; T4, thyroid hormone supplementation; TDC, thyroglossal duct cyst; TDCCa, thyroglossal duct cyst carcinoma (in this context, only differentiated thyroid gland cancer, such as papillary thyroid cancer or follicular thyroid cancer); TGC, thyroid gland cancer; TGN, thyroid gland nodule; TSH-Suppr., thyroid gland stimulating hormone suppression; TT, total thyroidectomy; US, ultrasound

The results of this analysis are illustrated in the treatment algorithm for differentiated thyroid carcinomas (particularly papillary thyroid carcinoma) in patients with TDCC (Fig. 3).

The optimal extent of surgical and adjuvant therapy for TDCCs remains widely debated in the literature. It is generally accepted that the Sistrunk procedure (SP), which involves resecting the cyst along with the central portion of the hyoid bone, should be the minimum surgical intervention. The findings reported by Patel et al. highlight that the extent of primary surgery significantly affects survival rates, while additional interventions such as total thyroidectomy (TT) or neck dissection (ND) do not influence outcomes [37]. Given the efficacy of SP in reducing recurrence rates [38], a “minimally invasive” approach is not disputed. However, the need for further surgical or adjuvant therapy beyond SP remains contentious. Some researchers argue for at least a TT, potentially with elective ND, due to the risk of secondary malignancies in the thyroid, even when preoperative findings are unremarkable [39]. In contrast, others argue that SP alone may suffice, particularly for patients with a normal thyroid and nodal status, especially in so-called “low-risk” cases [40, 41]. On the basis of the present data, we consider SP alone to be a safe first-line therapy for TDCC in patients without any risk factors. If a risk factor proves to be positive after removal of the cyst, the surgical therapy should be escalated by at least TT for the following RAI. Key risk factors include a history of radiation exposure, age over 45, positive family history, tumor size ≥ 2 cm, lymph-node or distant metastases, and histological risk factors as extracystic extension, lymph vessel or vein invasion, and high-risk mutations — e.g., *BRAF* V 600E, *TERT* promotor mutation [14, 26, 37, 42, 43].

Another fact that should be considered in the treatment of TDCC is that for PTC, the most common type of TDCC, simultaneous thyroid carcinomas were found in 34.6% of patients with additional TT — a rate approximately as high as is already known for multifocal carcinomas within the thyroid gland itself [44], but substantially lower than previous reports (up to 83.3%) in studies in which TT was routine [19, 45]. In the present cohort, 39.6% of patients with concurrent thyroid carcinoma had no detectable thyroid abnormalities preoperatively, and 47.4% of these were papillary thyroid carcinomas less than 1 cm (PTMC). Although these occult malignancies might have remained undetected without thyroidectomy, it is unclear whether their identification would affect survival outcomes, or if tumor progression was monitored during the follow-up. The role of PTMC has been debated internationally in relation to the extent of treatment. In Japan, where PTC is prevalent, active surveillance for PTMC has become more common, with > 50% of patients managed in this way [46]. Although surgical removal is the only cure, the low risk of progression (4.4% over 3.6 years) and low rates of locoregional (1%) and distant metastases (0.04%) during active surveillance warrant consideration of this approach, particularly for PTC in TDCC in patients with normal thyroid and nodal status. Complete thyroidectomy carries risks such as hypoparathyroidism, vocal cord paralysis, and postoperative bleeding [47], which may favor active surveillance in older patients (> 45 years). However, younger patients face double the progression risk, making surgical therapy preferable [48, 49].

In cases in which thyroid nodules are identified preoperatively, most authors recommend thyroidectomy along with SP [39]. In the present cohort, 41.2% of patients with thyroid abnormalities who underwent TT had concurrent thyroid carcinoma, indicating that more than half of the nodules were benign. The presence of thyroid nodules alone is therefore not a definitive criterion for thyroidectomy. Advanced ultrasound (US) detects thyroid nodules in 19–68% of individuals, particularly in iodine-deficient regions and among older patients, but thyroid carcinoma is far less common, with global incidence rates of 3.1/100,000 in men and 10.1/100,000 in women [50]. According to the American Thyroid Association (ATA) guidelines [51], thyroid nodules should be evaluated using US criteria, the ATA criteria, or the Thyroid Imaging Reporting and Data System (TI-RADS) classification, with fine-needle aspiration (FNA) if indicated. We do not recommend TT solely on the basis of TDCC presence; thorough preoperative evaluation, possibly including elastography and FNA, is advisable, especially in iodine-deficient regions and in older patients, in order to avoid unnecessary surgery and associated risks. Limited thyroid surgery, such as diagnostic lobectomy, should also be considered [52].

Neck dissection should be performed when preoperative lymph-node abnormalities are detected or in cases of concurrent thyroid carcinoma, as the risk of locoregional metastasis is greater. The present data show elevated rates of metastasis in the lateral compartment (15.0%) in comparison with the central compartment (5.6%) [29], supporting the recommendation for bilateral ND (levels IA–V) in addition to central ND (level VI) and total thyroidectomy [53–55].

The data on radioactive iodine (RAI) therapy in TDCC are limited, but guidelines for differentiated thyroid carcinoma from the ATA and national associations should guide its application [56]. RAI is recommended for tumors larger than 4 cm or with central or lateral lymph-node metastases. In the present cohort, 48.7% of patients received RAI treatment, following empirical dosage recommendations for differentiated thyroid carcinomas, although an individualized dose based on whole-body dosimetry may be considered [51].

For the rare squamous cell carcinomas (SCCs), given their more aggressive nature and greater likelihood of locoregional and distant metastases, elective bilateral ND in regions I–IV should be performed, even in clinically negative (cN0) necks. Thyroidectomy is generally unnecessary in these cases, and adjuvant radio(chemo)therapy should be considered in cases of positive nodal status or large tumors (≥ pT3), as well as in marginal or incomplete resections (R1), with a target dose of 60–66 Gy [57].

### Prognosis and follow-up

The prognosis for thyroglossal duct carcinoma (TDCC), especially for the common PTC subtype, is generally favorable, with a reported 10-year survival rate of 95–100%, surpassing the rate for primary thyroid carcinoma [7, 36]. In contrast, SCCs and PDTCs have a poorer prognosis, requiring more aggressive treatment, with the mortality from SCC reported at around 36% [14].

The present study confirms previous reports of a favorable prognosis for TDCC; however, the recurrence rate was higher at 7.4%, in comparison with 4.3% reported by Rayess et al. [7]. Tumor-related mortality remained low (0.95%) with an average follow-up of 41.5 months (95% CI, 45, 75; median 24 months). Distant metastases were rare (1.4%) and mainly associated with SCC, while locoregional metastases were higher at 20%. Notably, 15% of cases involved lateral cervical metastasis, and 5.6% had central compartment involvement, aligning with reports that lymphatic spread often occurs through the superior thyroid pedicle to the jugular chain [30].

Follow-up care should be adapted according to the tumor type: for PTC, thyroglobulin monitoring and whole-body scintigraphy are advised post-thyroidectomy, while CT imaging is essential for SCC. Routine clinical and ultrasonographic checks are recommended for all types in order to detect locoregional recurrences [58].

### Limitations and strengths

The primary limitations of this study relate to the retrospective nature of data collection — typical in rare conditions such as TDCCs, which primarily rely on case reports, small case series, and single-center studies. This approach introduces limitations, as the information available is often incomplete and of variable quality, with frequent gaps in critical details such as TNM staging, capsular invasion, follow-up, histology, and molecular markers.

Information on post-treatment complications is also limited. While a randomized controlled trial would provide stronger evidence, such studies are impractical for rare conditions. The systematic review component further introduces selection bias by including only published data; however, case reports with incomplete datasets were retained to reduce this bias, with the cohort adjusted accordingly for each analysis.

Despite these limitations, this study marks the second systematic review on TDCCs, incorporating the largest patient cohort to date and offering a basis for preliminary treatment recommendations.

## Conclusion

TDCCs are rare malignancies, PTC being the most common subtype. Despite their rarity, patients generally have a favorable prognosis, with a 10-year survival rate of 95–100%. Nevertheless, challenges persist regarding optimal diagnosis and treatment strategies, particularly concerning the extent of surgery and the need for adjuvant therapies. The Sistrunk procedure remains the standard initial treatment. However, additional interventions such as TT and ND should be considered, on the basis of tumor size (> pT2), nodal involvement (pN +), and patient-specific risk factors such as age, prior radiation in the head and neck region, positive family history, or high-risk histological subtypes (e.g., the tall-cell variant). Even the presence of mutations (*BRAF* V600E, *TERT* promotor mutation) could be a future indicator for high-risk patients. Further studies would be desirable. Concurrent thyroid nodules should be evaluated separately using the risk stratification systems recommended in the guidelines, as the risk of multifocal occurrence of PTC is approximately the same for TDCCs as for primary thyroid carcinomas.

Given the relatively low recurrence and metastasis rates, regular follow-up, including clinical assessments, ultrasonography, and additional imaging, when necessary, is essential for monitoring potential recurrences or metastases. Future studies are needed in order to refine the treatment guidelines further and enhance long-term management strategies for this rare but significant condition.

## Data Availability

All data supporting the findings of the systematic review of this study are available within the paper and its Supplementary Information. The data that support the findings of our own institution of this study are partially not openly available due to reasons of sensitivity and are available from the corresponding author upon reasonable request. Data are located in controlled access data storage at Friedrich Alexander University Erlangen-Nuremberg.

## Declarations

Consent was obtained from each patient for diagnostic procedures, therapeutic measures, and scientific data processing, all of which were approved by the university’s ethical review board. The study adhered to the university’s general contract conditions and the World Medical Association Declaration of Helsinki (as revised in 2013).
